# National Telehealth Contingency Staffing Program and Primary Care Quality in the VHA

**DOI:** 10.1001/jamanetworkopen.2024.53324

**Published:** 2025-01-07

**Authors:** Terrence Liu, Chelle L. Wheat, Jorge Rojas, Amy M. J. O’Shea, Karin M. Nelson, Ashok Reddy

**Affiliations:** 1 Center for Clinical Management Research, Ann Arbor Veterans Affairs (VA) Healthcare System, Ann Arbor, Michigan; 2 Institute for Healthcare Policy and Innovation, University of Michigan, Ann Arbor; 3 Division of General Medicine, Department of Internal Medicine, University of Michigan, Ann Arbor; 4 University of Michigan Medical School, Ann Arbor; 5 Center for Veteran-Centered and Value-Driven Care, Puget Sound VA Healthcare System, Seattle, Washington; 6 Veterans Affairs Center for Veteran-Centered and Value-Driven Care, Seattle, Washington; 7 Center for Access and Delivery Research and Evaluation, Iowa City VA Healthcare System, Iowa City; 8 Veterans Rural Health Resource Center, VA Office of Rural Health, Iowa City, Iowa; 9 Department of Internal Medicine, University of Iowa Carver College of Medicine, Iowa City; 10 Department of Health Systems and Population Health, University of Washington, Seattle; 11 Division of General Internal Medicine, Department of Medicine, University of Washington, Seattle

## Abstract

**Importance:**

The Veterans Health Administration (VHA) launched the Clinical Resource Hub (CRH), a national telehealth contingency staffing program, to address primary care staffing shortages and improve veteran access to primary care. How this large-scale telehealth intervention affects quality of care is unknown.

**Objective:**

To assess the quality of care for chronic disease management among US veterans receiving high vs low levels of CRH-delivered primary care services and whether racial and ethnic minority veterans experience outcomes similar to those of White veterans when receiving CRH-delivered care.

**Design, Setting, and Participants:**

This retrospective quality improvement study was conducted using VHA administrative data. Veterans receiving primary care services from CRH clinicians between October 1, 2022, to September 30, 2023, were included.

**Exposures:**

Low, medium, and high tertiles of CRH intensity, defined as the proportion of a veteran’s CRH primary care visits to their total primary care visits within the study period.

**Main Outcomes and Measures:**

The main outcomes were chronic disease quality measures for 2 common conditions in ambulatory care: diabetes and hypertension. Logistic regression models were used to estimate the association between individual-level receipt of care from a CRH clinician and the study outcomes.

**Results:**

This study included 71 508 veterans (mean [SD] age, 66 [15] years; 91.4% were male). Veterans who received a higher proportion of care from CRH clinicians were more likely to have improved blood pressure control compared with those who received a lower proportion of care. Higher vs lower CRH intensity was associated with improved blood pressure control among veterans with diabetes (79.5% [95% CI, 78.5%-80.4%] vs 76.6% [95% CI, 75.7%-77.5%]) and veterans with hypertension (76.8% [95% CI, 76.0%-77.5%] vs 73.9% [95% CI, 73.2%-74.7%]). Among racial and ethnic minority veterans, no association between CRH intensity and clinical quality was observed.

**Conclusions and Relevance:**

In this large retrospective cohort study among veterans receiving primary care services through a national telehealth contingency staffing program, veterans with a higher proportion of care from CRH clinicians had improvement in blood pressure outcomes. These findings suggest that the CRH program may be helpful in addressing veteran primary care needs without introducing or worsening disparities in ambulatory quality measures among racial and ethnic minority veterans. As health systems face staffing shortages in primary care, these findings can inform strategies for equitable implementation of large-scale telehealth initiatives to fill these gaps and maintain timely access to primary care.

## Key Points

**Question:**

Is participation in the Clinical Resource Hub (CRH) program, a national telehealth contingency staffing model, associated with quality of care among US veterans who lose their usual source of primary care?

**Findings:**

In this quality improvement study of 71 508 veterans, those who received a high vs low proportion of care from CRH clinicians were more likely to have improved blood pressure control. Among racial and ethnic minority veterans, no association between different levels of CRH-delivered care and clinical quality was observed.

**Meaning:**

These findings suggest that the CRH program may be helpful in addressing veteran primary care needs without introducing or worsening disparities in ambulatory quality measures among racial and ethnic minority veterans.

## Teaser

This quality improvement study examines the association of individual-level receipt of care from a Clinical Resource Hub clinician with outcomes for Veterans Health Administration (VHA) quality measures for diabetes and hypertension.

## Introduction

The national shortage of primary care physicians (PCPs) both within and outside the Veterans Health Administration (VHA) has presented substantial challenges for health systems to maintain timely health care access for patients.^1,2^ Staffing shortages can reduce patient access to health care and disrupt essential preventive and chronic disease services,^3^ which has been associated with increased mortality.^4^ Rural and underserved areas are particularly affected because patients living in these areas already face challenges in timely access to health care.^5^ Given the large proportion of veterans in rural areas, the VHA has prioritized expanding access to health care for rural veterans through initiatives such as telehealth.^6^

The Clinical Resource Hub (CRH) program is a national telehealth contingency staffing model that aims to address these types of shortages by providing clinical support to VHA facilities in rural and underserved areas.^7,8^ When a VHA clinic site loses staff or experiences an unpredicted deficit, the CRH program can provide care for patients at these sites. Using a hub-and-spoke care model, PCPs and support staff at 18 regional hub sites across the US can be deployed to spoke clinic sites. Clinical support is provided most often through telehealth modalities, including telephone- or video-based clinical services. Veterans may receive video-based telehealth either in their own home or in their local primary care clinic.^9^ Decisions regarding how and when to deploy the CRH hubs are made at the regional level and depend on local needs at each spoke site.

Studies evaluating the effects of large-scale telehealth interventions on primary care quality have been mixed. Telehealth can bridge geographic barriers, which may enhance access and improve ambulatory care quality. In a large study investigating health system–level telehealth use among Medicare fee-for-service beneficiaries, higher telehealth use was associated with decreased emergency department visits and increased adherence to metformin and statins.^10^ However, clinical care that requires physical examination may be inadequately addressed by telehealth, which may lead to wasteful or low-quality care. Some studies have reported that hospitalizations for ambulatory care–sensitive conditions—which are acute or chronic health issues that lead to potentially preventable hospitalizations when not treated in the outpatient primary care setting and are often used as an indicator for primary care access and quality—were higher among health systems using a high compared with a low volume of telehealth.^11,12^ Furthermore, disparities in telehealth use among rural, older, and historically underrepresented racial and ethnic minority patients have been previously described.^13,14,15^ Given that telehealth interventions (eg, videoconferencing, remote patient monitoring, and text messaging) may introduce or exacerbate existing disparities, evaluating whether such interventions affect vulnerable patients differently must be taken into account.

Although telehealth can help increase access to care, how it affects quality of care in the context of individuals losing their usual source of primary care is incompletely described. Previous studies suggest that the CRH program can help veterans maintain access to care in the setting of clinician and staff turnover,^16^ although questions remain regarding how a greater proportion of telehealth-delivered care by clinicians other than a veteran’s usual PCP may affect quality of care at the patient level. Few studies of large-scale telehealth interventions focus on individual-level measures, which may be more informative for quality-of-care metrics, compared with aggregate-level measures.^17,18^ Previous studies have reported telehealth exposure associated with higher clinical quality; however, results were limited to a single regional health system and did not account for differing telehealth exposure levels among patients.^19^

As a large-scale national telehealth contingency staffing intervention, the CRH program provides an opportunity to study how telehealth affects primary care quality. In this study, we investigated the association of individual-level receipt of care from a CRH clinician with outcomes in chronic disease quality measures for 2 common conditions in ambulatory care: diabetes and hypertension. We hypothesized that veterans who receive a higher proportion of primary care visits with CRH clinicians would have higher performance on quality measures and that the program would have similar outcomes among racial and ethnic minority veterans.

## Methods

We conducted this retrospective quality improvement study of veterans who received CRH primary care services between October 1, 2022, and September 30, 2023, to examine the association between the proportion of CRH-delivered care and clinical quality measures. The evaluation efforts were part of an ongoing quality improvement effort at the VHA and were not considered human participant research activity as determined by the VA Office of Primary Care; thus, this study was not subject to institutional review board review and informed consent was waived. The study followed the Strengthening the Reporting of Observational Studies in Epidemiology (STROBE) and the Standards for Quality Improvement Reporting Excellence (SQUIRE) reporting guidelines.

### Data Source and Study Cohort

The data for this study were obtained from the VHA Corporate Data Warehouse, a national repository of clinical and administrative data from the VHA electronic health record system. Data from the Electronic Quality Measurement (eQM) platform from September 2023 were extracted.

To identify veterans regularly receiving primary care services, we limited our cohort to veterans who had a minimum of 3 primary care visits in the study period, with at least 1 of those visits being a CRH primary care visit. We investigated clinical quality measures in diabetes and hypertension, 2 common conditions treated in primary care. We constructed 2 separate cohorts for analysis, which included veterans with diagnoses of diabetes and hypertension, respectively. The subgroup of veterans with comorbid diabetes and hypertension diagnoses was included in both cohorts for analyses.

### Definition of CRH Intensity

We use the term *CRH intensity* to represent the individual-level receipt of primary care from a CRH clinician, which was measured by the proportion of CRH-delivered primary care visits relative to total primary care visits during the study period. We created data-driven tertiles of CRH intensity, with the 3 groups representing low, medium, and high intensity. Veterans with low CRH intensity were used as the reference group for all analyses.

### Outcome Measures

The VHA tracks performance through quality measures based on the Centers for Medicare & Medicaid Services Healthcare Effectiveness Data and Information Set using the eQM platform.^20^ The specific outcome measures in our study were selected on the basis of covering high-priority primary care conditions, being associated with patient outcomes, and being well established inside and outside of the VHA as core measures of primary care clinical quality.^21^ In this study, diabetes measures included annual measurement of hemoglobin A_1c_ (HbA_1c_), diabetic nephropathy screening, statin therapy for diabetes, statin adherence for diabetes, and poorly controlled HbA_1c_. Hypertension measures included well-controlled blood pressure among veterans with diabetes and well-controlled blood pressure among veterans with hypertension. eTable 1 in Supplement 1 outlines all quality measurement outcomes used in this study.

### Covariates

Veteran characteristics evaluated included the following: age (in years), sex (male or female), race and ethnicity^22^ (American Indian or Alaska Native, Asian or Native Hawaiian or Other Pacific Islander, Hispanic, non-Hispanic Black [hereinafter, Black], non-Hispanic White [hereinafter, White], other race or ethnicity, or multiple races or ethnicities), marital status (married or other), rurality (urban, rural, highly rural, or insular islands), driving distance to the nearest VHA primary care clinic, total number of primary care visits, Gagne comorbidity score,^23^ Nosos comorbidity score,^24,25^ and neighborhood socioeconomic status (SES) index.^26^ Data on race and ethnicity were collected through a previously developed algorithm that aggregates values across multiple VA medical records and demographic datasets, prioritizing sources of patient self-report; categories of other race or ethnicity are included from historical response options in some datasets and include “declined,” “multiple,” or “unknown.”^22^ These data were included to assess for telehealth-related health disparities. Covariates were obtained from administrative databases, and the value from the beginning of the study period was selected.

### Statistical Analysis

We conducted individual-level analyses using logistic regression modeling to estimate associations between CRH intensity and clinical quality measures in diabetes and hypertension. Because several factors may influence both the exposure (CRH intensity) and the outcome (clinical quality measures), we included age, sex, race and ethnicity, marital status, rurality, driving distance to the nearest VHA primary care clinic, total number of primary care visits, comorbidity scores, and SES index in our models to account for potential confounders. Importantly, we included in our model the total number of primary care visits during the study period as a confounder, because veterans with more visits have more opportunities to complete quality measures related to diabetes and hypertension. Outcomes are presented as the estimated probability^27,28^ of meeting the criteria for the corresponding quality measure at the end of the study period. Missing data values for all covariate measures were excluded from the analysis.

To investigate whether CRH intensity affected groups of veterans differently, particularly racial and ethnic minority populations, we repeated our analyses with an interaction term between the main estimator variable, CRH intensity, and the race and ethnicity covariate. We applied a Bonferroni correction to account for multiple hypothesis testing. Racial and ethnic minority status was determined based on administrative race and ethnicity records; racial and ethnic minority veterans were those not recorded as being White.

A threshold of *P* < .05 (2-tailed) was used to assess statistical significance. All statistical analyses were performed using Stata, version 18 (StataCorp LLC).

## Results

### Patient Characteristics

Our cohort included 71 508 veterans (mean [SD] age, 66 [15] years; 91.4% were male and 8.6% were female). Race and ethnicity was available for 70 525 of 71 508 veterans; 1.1% were American Indian or Alaska Native, 2.5% were Asian or Native Hawaiian or Other Pacific Islander, 14.8% were Black, 5.2% were Hispanic, 72.8% were White, and 2.3% were of other race or ethnicity or multiple races or ethnicities. There were 3589 veterans (5.0%) with only diabetes, 26 227 (36.7%) with only hypertension, and 41 692 (58.3%) with both diabetes and hypertension. The mean (SD) percentage of veterans with low, medium, and high CRH intensity was 13.8% (4.8%), 30.7% (5.1%), and 69.1% (18.1%), respectively (Table). Veterans across the different CRH intensity groups had similar age, sex, and race and ethnicity distributions (Table). Veterans with high CRH intensity tended to live in rural areas compared with veterans with low CRH intensity (47.9% vs 40.1%; Table). Additionally, veterans with high CRH intensity tended to have lower mean (SD) comorbidity scores compared with veterans with low CRH intensity for both the Gagne (0.4 [1.3] vs 0.8 [1.8]) and Nosos (1.1 [0.7] vs 1.4 [0.9]) measures (Table). These distributions in the entire cohort were similar to those observed in the diabetes (eTable 2 in Supplement 1) and hypertension (eTable 3 in Supplement 1) cohorts.

**Table.** Demographic and Health Characteristics of Veterans Receiving Low, Medium, and High CRH Intensity Levels of Care^a^

Characteristic	CRH intensity tertile (N = 71 508)			
	Low (n = 23 857 [33.4%])	Medium (n = 25 547 [35.7%])	High (n = 22 104 [30.9%])	*P* value
CRH intensity, mean (SD), %	13.8 (4.8)	30.7 (5.1)	69.1 (18.1)	<.001
Age, mean (SD), y	67 (14)	65 (15)	65 (15)	<.001
Sex				
Male	21 353 (90.4)	22 994 (91.2)	20 251 (92.6)	<.001
Female	2280 (9.6)	2220 (8.8)	1613 (7.4)	
Race and ethnicity^b^				
American Indian or Alaska Native	266 (1.1)	291 (1.2)	223 (1.0)	<.001
Asian or Native Hawaiian or Other Pacific Islander	547 (2.3)	606 (2.4)	647 (3.0)	
Black	3565 (15.1)	3507 (13.9)	3478 (15.9)	
Hispanic	1200 (5.1)	1348 (5.4)	1198 (5.5)	
White	17 435 (74.0)	18 849 (75.0)	15 740 (72.1)	
Other race or ethnicity or multiple races or ethnicities^c^	554 (2.4)	541 (2.2)	530 (2.4)	
Marital status				
Not married	10 704 (45.3)	10 734 (42.7)	8862 (40.7)	<.001
Married	12 900 (54.7)	14 424 (57.3)	12 924 (59.3)	
Rurality				
Urban	12 723 (53.9)	13 188 (52.4)	9837 (45.0)	<.001
Rural	9457 (40.1)	10 401 (41.3)	10 470 (47.9)	
Highly rural or insular islands	1415 (6.0)	1594 (6.3)	1536 (7.0)	
Driving distance to PCC, mean (SD), miles^d^	18 (20)	19 (23)	22 (28)	<.001
No. of primary care visits, mean (SD)	10 (6)	6 (4)	6 (4)	<.001
Gagne comorbidity score, mean (SD)^e^	0.8 (1.8)	0.5 (1.4)	0.4 (1.3)	<.001
Nosos comorbidity score, mean (SD)^f^	1.4 (0.9)	1.2 (0.8)	1.1 (0.7)	<.001
Neighborhood SES index, decile^g^				
0	1485 (6.7)	1495 (6.4)	1277 (6.4)	<.001
1	2199 (9.9)	2142 (9.1)	2118 (10.5)	
2	2789 (12.5)	2841 (12.1)	2859 (14.2)	
3	2767 (12.4)	2938 (12.5)	2850 (14.2)	
4	2807 (12.6)	3002 (12.8)	2780 (13.8)	
5	2795 (12.6)	2949 (12.6)	2534 (12.6)	
6	2418 (10.9)	2636 (11.3)	1955 (9.7)	
7	2237 (10.1)	2404 (10.3)	1733 (8.6)	
8	1786 (8.0)	1893 (8.1)	1262 (6.3)	
9	974 (4.4)	1140 (4.9)	732 (3.6)	

Abbreviations: CRH, Clinical Resource Hub; PCC, primary care clinic; SES, socioeconomic status.

^a^ Unless specified otherwise, values are presented as No. (%) of veterans. Values may not sum to 100% due to missing data, which are not shown.

^b^ Based on race and ethnicity categories developed by Hernandez et al.^22^

^c^ Categories of other race are included from historical response options in some datasets and include “declined,” “multiple,” or “unknown.”

^d^ To convert miles to kilometers, multiply by 1.6.

^e^ Scores range from less than 0 to greater than 9, with increased scores corresponding to increased risk of 1-year mortality.^23^

^f^ Scores are centered around 1, which indicates that the patient is expected to have costs that are the national average for Veterans Affairs (VA) patients. If a patient has a risk score of 2.5, then the patient has an expected cost that is 2.5 times higher than the average VA patient.^24,25^

^g^ Reported as deciles based on US Census data, as a surrogate marker for income.^26^

### Association of CRH Intensity With Outcome Measures

#### Diabetes Quality Measures

**Figure 1.**
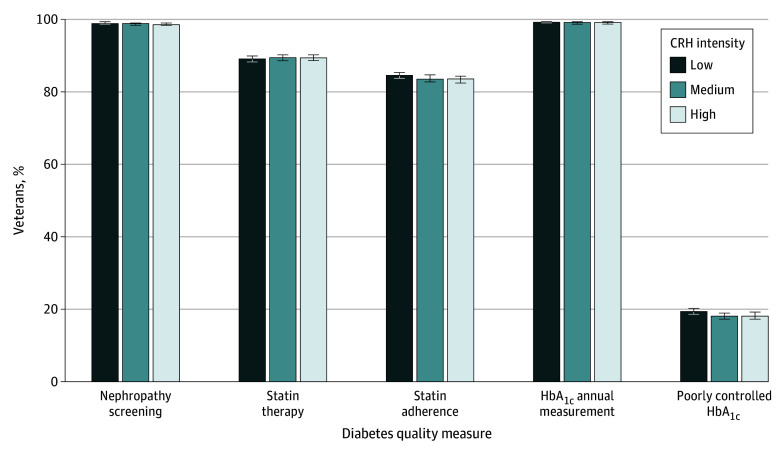
Estimated Probabilities of Diabetes Quality Measures by Clinical Resource Hub (CRH) Intensity The following diabetes quality measures were assessed for veterans: nephropathy screening (documented within the measurement year), statin therapy (≥1 dispensing of a statin of any intensity within the measurement year), statin adherence (statin prescribed for 80% of the treatment period within the measurement year), hemoglobin A_1c_ (HbA_1c_) (documented annual measurement), and poorly controlled HbA_1c_ (most recent HbA_1c_ >9% or no evidence of testing within the measurement year). Models were adjusted for age, sex, race and ethnicity, marital status, rurality, driving distance to the nearest Veterans Health Administration primary care clinic, number of primary care visits, comorbidity scores, and socioeconomic status index. Error bars indicate 95% CIs.

Estimated probabilities of veterans having completed diabetes quality measures were similar across all CRH intensity levels (Figure 1). There was no association between CRH intensity and diabetes quality measures (Figure 1 and eTable 4 in Supplement 1). Full regression results are presented in eTable 5 in Supplement 1.

#### Hypertension Quality Measures

**Figure 2.**
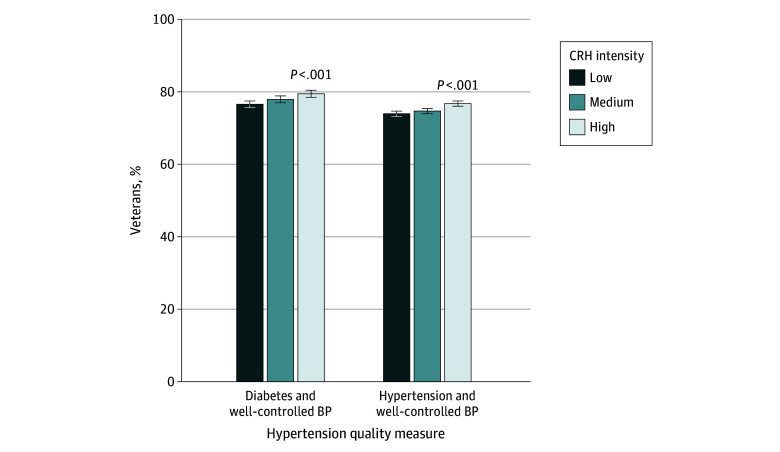
Estimated Probabilities of Hypertension Quality Measures by Clinical Resource Hub (CRH) Intensity The following diabetes quality measures were assessed for veterans: diabetes with well-controlled blood pressure (BP) (defined as most recent recorded BP <140/90 mm Hg) or hypertension with well-controlled BP. Models were adjusted for age, sex, race and ethnicity, marital status, rurality, driving distance to the nearest Veterans Health Administration primary care clinic, number of primary care visits, comorbidity scores, and socioeconomic status index. Error bars indicate 95% CIs.

High CRH intensity was associated with improved blood pressure control outcomes (Figure 2 and eTable 4 in Supplement 1). Among veterans with diabetes, high CRH intensity was associated with improved blood pressure (79.5% [95% CI, 78.5%-80.4%] vs 76.6% [95% CI, 75.7%-77.5%]) compared with low CRH intensity. This finding was also true among veterans with hypertension (high vs low CRH intensity: 76.8% [95% CI, 76.0%-77.5%] vs 73.9% [95% CI, 73.2%-74.7%]). Full regression results are presented in eTable 6 in Supplement 1.

#### Clinical Quality Measures Among Racial and Ethnic Minority Veterans

**Figure 3.**
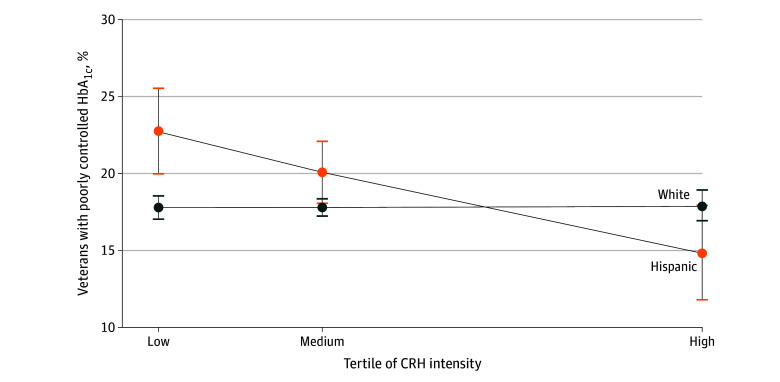
Estimated Probability of Veterans Health Administration Clinical Resource Hub (CRH) Intensity Interaction With Race and Ethnicity for Poorly Controlled Hemoglobin A_1c_ (HbA_1c_) Poorly controlled HbA_1c_ was defined as a most recent HbA_1c_ measurement greater than 9% or no evidence of testing within the measurement year. Models were adjusted for age, sex, marital status, rurality, driving distance to the nearest Veterans Health Administration primary care clinic, number of primary care visits, comorbidity scores, and socioeconomic status index. Race and ethnicity was treated as an interaction term with CRH intensity. Bonferroni correction was applied to account for multiple hypothesis testing. Error bars indicate 95% CIs.

Overall, we did not observe associations between clinical quality outcomes and interaction of race and ethnicity with CRH intensity. However, high CRH intensity was associated with lower estimated probability of poor glycemic control among Hispanic veterans compared with White veterans (Figure 3).

## Discussion

In this large retrospective cohort study of US veterans receiving primary care services through a national telehealth contingency staffing program, receiving higher levels of primary care from a CRH clinician was associated with improvements in blood pressure control. We did not observe associations between CRH intensity and the diabetes outcomes studied. Among racial and ethnic minority veterans, we found that higher levels of CRH intensity were not associated with changes in most quality measures studied.

Improvement in blood pressure control among veterans who received higher levels of CRH may reflect the ability for telehealth to facilitate the virtual management of hypertension. In a large pragmatic trial that compared telehealth-based care vs clinic-based care for uncontrolled hypertension, systolic blood pressure decreased substantially by a similar amount in both groups.^29^ Notably, patients who received telehealth-based care were more likely to self-monitor their home blood pressure and report higher satisfaction with their care compared with those who received clinic-based care. Although our data do not include patient-reported outcomes, the added convenience of telehealth-enabled home blood pressure readings could represent a potential mechanism of how telehealth facilitates management of hypertension. Our findings add to the literature of telehealth as an effective modality of care for managing hypertension compared with usual clinic-based care.^30^

It is important to note that our data were limited in helping identify the precise mechanism of improved blood pressure control with higher levels of CRH intensity. Future studies should incorporate additional hypertension measures to explore ways in which telehealth facilitates hypertension management, whether that involves new prescriptions of antihypertensive medications, improved adherence to these medications, or behavioral modifications.

In contrast to our findings with high CRH intensity and improved blood pressure control, we did not observe an association between CRH intensity or any of our diabetes-related outcomes. This lack of association might be due to differences in how hypertension and diabetes are monitored. For hypertension, patients are often encouraged to measure their blood pressure at home, given the concern of potential misleading estimates of blood pressure measured in the clinic.^31^ For diabetes, an HbA_1c_ laboratory test is considered the standard of care for assessing glycemic control.^32^ Whereas telehealth can facilitate patients sharing their home blood pressure readings with their clinician for hypertension management, the benefits of a virtual visit might be more limited when diabetes management relies on testing that necessitates an in-person laboratory visit. Our findings may reflect the limits of telehealth in managing certain chronic conditions that require some degree of in-person laboratory testing or monitoring. Although our study does not capture whether veterans shared home blood pressure measurements with their clinician during their CRH telehealth visit, the ability to obtain point-of-care blood pressure readings during a virtual visit to inform treatment decisions could potentially make hypertension management more amenable to telehealth compared with diabetes management.

Among racial and ethnic minority veterans, we found no association between CRH intensity and most clinical quality outcomes studied. The CRH program was intended to support clinics that may be disproportionately affected by unexpected staffing shortages, particularly clinics in underserved or rural areas. Racial and ethnic minority veterans may be more disproportionately affected if they reside in such areas already facing steep challenges in health care access. Given the potential harms of telehealth interventions introducing or exacerbating health disparities, it is encouraging that high CRH intensity was not associated with poorer clinical outcomes in this study. Although further research is needed to explore mechanisms of how a higher proportion of primary care services delivered by CRH clinicians may benefit certain groups of racial and ethnic minority veterans, our results help inform how health systems can equitably implement large-scale telehealth initiatives in primary care.

### Limitations

We acknowledge several limitations of our study. First, it was performed among veterans receiving care within the VHA and may not be generalizable to other populations. Second, it focused on veterans who received CRH-delivered care; we did not evaluate quality measures among veterans who did not receive CRH-delivered care, which limits comparisons of CRH-delivered care to usual ambulatory care. However, we intentionally limited our study to this cohort to investigate outcomes of telehealth-delivered primary care in the context of veterans losing their usual source of primary care. Third, the study was limited to 1 year, and a longer time frame may be needed to evaluate the longitudinal effects of the CRH program on outcomes in ambulatory quality of care, particularly outcomes such as optimal glycemic or blood pressure control.

## Conclusions

In this quality improvement study of veterans receiving primary care services through a national telehealth contingency staffing program, veterans receiving a higher proportion of care from CRH clinicians had improvement in blood pressure outcomes. Despite the study limitations, our findings improve our understanding of the association of a national telehealth contingency staffing program with individual health outcomes in primary care. Our findings of improvements in blood pressure control outcomes suggest that the CRH program may be helpful in addressing important primary care needs of veterans through telehealth when clinician turnover occurs. That the implementation of CRH did not introduce or exacerbate disparities for racial and ethnic minority veterans provides insights on ways to equitably deliver primary care services within large integrated health systems. Because health systems both within and outside the VHA face staffing challenges in primary care, our findings should inform large-scale implementation strategies to strengthen the primary care workforce and equitably provide high-quality primary care services.

## Notes

Supplement 1. **eTable 1.** Primary Care Chronic and Preventive Disease Electronic Quality Measurements (eQMs) **eTable 2.** Demographic and Health Characteristics of Veterans With Diabetes Receiving Low, Medium, and High Clinical Resource Hub Intensity (CRH) Levels of Care **eTable 3.** Demographic and Health Characteristics of Veterans With Hypertension Receiving Low, Medium, and High Clinical Resource Hub Intensity (CRH) Levels of Care **eTable 4.** Estimated Probabilities of Veteran Performance on Diabetes and Hypertension Quality Measures by Clinical Resource Hub (CRH) Intensity **eTable 5.** Multivariable Regression Results of Association Between Clinical Resource Hub (CRH) Intensity and Diabetes Quality Measures **eTable 6.** Multivariable Regression Results of Association Between Clinical Resource Hub (CRH) Intensity and Hypertension Quality Measures
eReferences

Supplement 2.
Data Sharing Statement
