# Supplementary material for: National Telehealth Contingency Staffing Program and Primary Care Quality in the VHA
Source: JAMA Netw Open. 2025 Jan 7;8(1):e2453324. doi: 10.1001/jamanetworkopen.2024.53324 (PMC11707631; doi:10.1001/jamanetworkopen.2024.53324)
Supplement: Supplement 1. — eTable 1. Primary Care Chronic and Preventive Disease Electronic Quality Measurements (eQMs) eTable 2. Demographic and Health Characteristics of Veterans With Diabetes Receiving Low, Medium, and High Clinical Resource Hub Intensity (CRH) Levels of Care eTable 3. Demographic and Health Characteristics of Veterans With Hypertension Receiving Low, Medium, and High Clinical Resource Hub Intensity (CRH) Levels of Care eTable 4. Estimated Probabilities of Veteran Performance on Diabetes and Hypertension Quality Measures by Clinical Resource Hub (CRH) Intensity eTable 5. Multivariable Regression Results of Association Between Clinical Resource Hub (CRH) Intensity and Diabetes Quality Measures eTable 6. Multivariable Regression Results of Association Between Clinical Resource Hub (CRH) Intensity and Hypertension Quality Measures eReferences [file jamanetwopen-e2453324-s001.pdf]

Supplementary Online Content

Liu T, Wheat CL, Rojas J, O'Shea AMJ, Nelson KM, Reddy A. National telehealth contingency staffing program and primary care quality in the VHA. *JAMA Netw Open*. 2025;8(1):e2453324. doi:10.1001/jamanetworkopen.2024.53324

eTable 1. Primary Care Chronic and Preventive Disease Electronic Quality Measurements (eQMs)

eTable 2. Demographic and Health Characteristics of Veterans With Diabetes Receiving Low, Medium, and High Clinical Resource Hub Intensity (CRH) Levels of Care

eTable 3. Demographic and Health Characteristics of Veterans With Hypertension Receiving Low, Medium, and High Clinical Resource Hub Intensity (CRH) Levels of Care

eTable 4. Estimated Probabilities of Veteran Performance on Diabetes and Hypertension Quality Measures by Clinical Resource Hub (CRH) Intensity

eTable 5. Multivariable Regression Results of Association Between Clinical Resource Hub (CRH) Intensity and Diabetes Quality Measures

eTable 6. Multivariable Regression Results of Association Between Clinical Resource Hub (CRH) Intensity and Hypertension Quality Measures

eReferences

This supplementary material has been provided by the authors to give readers additional information about their work.

© 2025 Liu T et al. *JAMA Network Open.*

eTable 1. Primary Care Chronic and Preventive Disease Electronic Quality Measurements (eQMs)

	Numerator	Denominator	Preferred Score Direction
Diabetes Quality Measures			
Nephropathy Screening	Veterans with documented screening for nephropathy within measurement year	Veterans 18-75 years old with a diagnosis of DM	Higher is better
Statin Therapy	Veterans with at least one dispensing of statin of any intensity within measurement year	Veterans 40-75 years old with a diagnosis of DM	Higher is better
Statin Adherence	Veterans who have a statin prescribed for 80% of treatment period within measurement year	Veterans 40-75 years old with a diagnosis of DM	Higher is better
HgbA1c Annual Measurement	Veterans with documentation of annual measurement of HgbA1c	Veterans 18-75 years old with a diagnosis of DM	Higher is better
HgbA1c, poorly controlled	Veterans with most recent HgbA1c greater than 9 or no evidence of test within measurement year	Veterans 18-75 years old with a diagnosis of DM	Lower is better
Hypertension Quality Measures			
BP, well controlled (DM[a])	Veterans with most recent recorded BP as < 140/90mmHg	Veterans 18-75 years old with a diagnosis of DM	Higher is better
BP, well controlled (HTN[b])	Veterans with most recent recorded BP as < 140/90mmHg	Veterans 18-85 years old with a diagnosis of hypertension	Higher is better

Abbreviations: HgbA1c, Hemoglobin A1c; BP, Blood pressure; DM, diabetes mellitus; HTN, hypertension

[a] The diabetes cohort consists of Veterans with only diabetes as well as comorbid diabetes and hypertension

[b] The hypertension cohort consists of Veterans with only hypertension as well as comorbid hypertension and diabetes

© 2025 Liu T et al. *JAMA Network Open.*

eTable 2. Demographic and Health Characteristics of Veterans With Diabetes[a] Receiving Low, Medium, and High Clinical Resource Hub (CRH) Intensity Levels of Care

	CRH Intensity			
	Low	Medium	High	p-value
N	9,967 (39.3%)	8,020 (31.6%)	7,369 (29.1%)	
CRH Intensity, mean (SD)	13.1 (4.9)	30.6 (5.3)	69.0 (18.4)	
Age, mean (SD), years	68 (9)	68 (10)	68 (10)	<0.001
Sex				
Male	9,338 (93.7%)	7,579 (94.5%)	7,025 (95.3%)	<0.001
Female	629 (6.3%)	441 (5.5%)	344 (4.7%)	
Race and ethnicity[b]				
Non-Hispanic White	7,115 (71.5%)	5,640 (70.5%)	4,987 (67.8%)	<0.001
Non-Hispanic Black	1,665 (16.7%)	1,386 (17.3%)	1,348 (18.3%)	
Hispanic	561 (5.6%)	469 (5.9%)	419 (5.7%)	
Asian/Pacific Islander/Native Hawaiian	233 (2.3%)	199 (2.5%)	299 (4.1%)	
American Indian/Alaska Native	119 (1.2%)	111 (1.4%)	92 (1.3%)	
Multiracial or other	254 (2.6%)	190 (2.4%)	208 (2.8%)	
Marital Status				
Not Married	4,242 (42.6%)	3,272 (40.8%)	2,829 (38.5%)	<0.001
Married	5,714 (57.4%)	4,740 (59.2%)	4,527 (61.5%)	
Rurality				
Urban	5,371 (53.9%)	4,147 (51.7%)	3,295 (44.7%)	<0.001
Rural	3,971 (39.9%)	3,391 (42.3%)	3,514 (47.7%)	
Highly rural or insular islands	620 (6.2%)	481 (6.0%)	556 (7.5%)	
Drive distance to PCC, mean (SD), miles	18 (20)	19 (22)	22 (27)	<0.001
Number of primary care visits, mean (SD)	11 (7)	7 (5)	7 (5)	<0.001
Gagne Comorbidity Score[c], mean (SD)	1.1 (1.9)	0.8 (1.7)	0.6 (1.5)	<0.001
Nosos Comorbidity Score[d], mean (SD)	1.7 (1.1)	1.5 (0.9)	1.4 (0.8)	<0.001
Neighborhood SES Index[e] (decile)				
0	703 (7.3%)	532 (7.7%)	588 (7.5%)	<0.001
1	994 (10.3%)	742 (10.8%)	916 (11.7%)	
2	1,297 (13.5%)	865 (12.6%)	1,164 (14.8%)	
3	1,203 (12.5%)	878 (12.7%)	1,099 (13.9%)	
4	1,246 (12.9%)	886 (12.9%)	1,085 (13.8%)	
5	1,169 (12.2%)	829 (12.0%)	958 (12.2%)	
6	991 (10.3%)	746 (10.8%)	730 (9.3%)	
7	918 (9.6%)	645 (9.4%)	625 (7.9%)	
8	697 (7.3%)	504 (7.3%)	452 (5.8%)	
9	392 (4.1%)	264 (3.8%)	247 (3.1%)	

Abbreviations: PCC, primary care clinic; SES, socioeconomic status

[a] The diabetes cohort consists of Veterans with only diabetes as well as comorbid diabetes and hypertension

[b] Based on race and ethnicity categories developed by Hernandez et al[1]

[c] Scores range from <0 to >9, with increased scores corresponding to increased risk of 1-year mortality[2]

[d] The Nosos scores are centered around 1, which indicates the patient is expected to have costs that are the national average for VA patients. If a patient has a risk score of 2.5, then the patient has an expected cost that is 2.5 times higher than the average VA patient.[3,4]

[e] Neighborhood socioeconomic status is reported as deciles based on census data, as a surrogate marker for income[5]

© 2025 Liu T et al. *JAMA Network Open.*

eTable 3. Demographic and Health Characteristics of Veterans With Hypertension[a] Receiving Low, Medium, and High Clinical Resource Hub (CRH) Intensity Levels of Care

	CRH Intensity			
	Low	Medium	High	p-value
N	17,003 (35.4%)	16,348 (34.1%)	14,643 (30.5%)	
CRH Intensity, mean (SD)	13.6 (4.8)	30.7 (5.2)	69.0 (18.2)	
Age, mean (SD), years	70 (11)	69 (11)	69 (11.3)	<0.001
Sex				
Male	15,937 (93.7%)	15,513 (94.9%)	13,934 (95.2%)	<0.001
Female	1,066 (6.3%)	835 (5.1%)	709 (4.8%)	
Race and ethnicity[b]				
Non-Hispanic White	12,640 (74.5%)	12,272 (75.3%)	10,505 (71.9%)	<0.001
Non-Hispanic Black	2,634 (15.5%)	2,404 (14.7%)	2,538 (17.4%)	
Hispanic	780 (4.6%)	752 (4.6%)	673 (4.6%)	
Asian/Pacific Islander/Native Hawaiian	340 (2.0%)	352 (2.2%)	430 (2.9%)	
American Indian/Alaska Native	192 (1.1%)	178 (1.1%)	145 (1.0%)	
Multiracial or other	381 (2.2%)	350 (2.1%)	325 (2.2%)	
Marital Status				
Not Married	7,338 (43.2%)	6,617 (40.5%)	5,677 (38.9%)	<0.001
Married	9,647 (56.8%)	9,709 (59.5%)	8,935 (61.1%)	
Rurality				
Urban	8,896 (52.3%)	8,200 (50.2%)	6,312 (43.1%)	<0.001
Rural	7,036 (41.4%)	7,058 (43.2%)	7,233 (49.4%)	
Highly rural or insular islands	1,065 (6.3%)	1,086 (6.6%)	1,090 (7.4%)	
Drive distance to PCC, mean (SD), miles	20 (20)	20 (23)	22 (28)	<0.001
Number of primary care visits, mean (SD)	11 (6)	6 (4)	6 (4)	<0.001
Gagne Comorbidity Score[c], mean (SD)	0.9 (1.8)	0.5 (1.5)	0.4 (1.3)	<0.001
Nosos Comorbidity Score[d], mean (SD)	1.5 (0.9)	1.3 (0.8)	1.2(0.7)	<0.001
Neighborhood SES Index[e] (decile)				
0	1,175 (7.2%)	1,058 (6.7%)	954 (6.9%)	<0.001
1	1,626 (9.9%)	1,505 (9.6%)	1,584 (11.4%)	
2	2,131 (13.0%)	1,994 (12.7%)	2,034 (14.7%)	
3	2,068 (12.6%)	2,047 (13.0%)	1,995 (14.4%)	
4	2,073 (12.7%)	2,046 (13.0%)	1,961 (14.1%)	
5	2,072 (12.7%)	1,921 (12.2%)	1,723 (12.4%)	
6	1,714 (10.5%)	1,768 (11.2%)	1,296 (9.3%)	
7	1,599 (9.8%)	1,532 (9.7%)	1,115 (8.0%)	
8	1,251 (7.7%)	1,204 (7.7%)	781 (5.6%)	
9	649 (3.9%)	652 (4.1%)	431 (3.1%)	

Abbreviations: PCC, primary care clinic; SES, socioeconomic status

[a] The hypertension cohort consists of Veterans with only hypertension as well as comorbid hypertension and diabetes

[b] Based on race and ethnicity categories developed by Hernandez et al[1]

[c] Scores range from <0 to >9, with increased scores corresponding to increased risk of 1-year mortality[2]

[d] The Nosos scores are centered around 1, which indicates the patient is expected to have costs that are the national average for VA patients. If a patient has a risk score of 2.5, then the patient has an expected cost that is 2.5 times higher than the average VA patient.[3,4]

[e] Neighborhood socioeconomic status is reported as deciles based on census data, as a surrogate marker for income[5]

© 2025 Liu T et al. *JAMA Network Open.*

eTable 4. Estimated Probabilities of Veteran Performance on Diabetes and Hypertension Quality Measures by Clinical Resource Hub (CRH) Intensity

	CRH Intensity		
	Low<br>AME (95% CI)	Medium<br>AME (95% CI)	High<br>AME (95% CI)
Diabetes Quality Measures, %			
Nephropathy Screening[a]	98.8 (98.6 - 99.1)	98.6 (98.3 - 98.9)	98.6 (98.3 - 98.8)
Statin Therapy[b]	88.9 (88.2 - 89.7)	89.4 (88.6 - 90.1)	89.4 (88.6 - 90.1)
Statin Adherence[c]	84.5 (83.6 - 85.3)	83.5 (82.5 - 84.4)	83.4 (82.4 - 84.4)
HgbA1c Annual Measurement[d]	99.0 (98.8 - 99.3)	98.9 (98.7 - 99.2)	98.9 (98.7 - 99.2)
HgbA1c, poorly controlled[e]	19.3 (18.5 - 20.1)	18.0 (17.2 - 18.9)	18.1 (17.2 - 19.0)
Hypertension Quality Measures, %			
BP, well controlled (DM)[f]	76.6 (75.7 - 77.5)	77.9 (77.0 - 78.9)	79.5 (78.5 - 80.4)
BP, well controlled (HTN)[g]	73.9 (73.2 - 74.7)	74.7 (74.9 - 75.4)	76.8 (76.0 - 77.5)

Abbreviations: AME, Average Marginal Effect; HgbA1c, Hemoglobin A1c; BP, Blood pressure; DM, diabetes mellitus; HTN, hypertension

[a] Veterans with documented screening for nephropathy within measurement year
[b] Veterans with at least one dispensing of statin of any intensity within measurement year
[c] Veterans who have a statin prescribed for 80% of treatment period within measurement year
[d] Veterans with documentation of annual measurement of HgbA1c
[e] Veterans with most recent HgbA1c greater than 9 or no evidence of test within measurement year
[f] Veterans with diabetes mellitus with most recent recorded BP as < 140/90mmHg
[g] Veterans with hypertension with most recent recorded BP as < 140/90mmHg

© 2025 Liu T et al. *JAMA Network Open.*

eTable 5. Multivariable Regression Results of Association Between Clinical Resource Hub (CRH) Intensity and Diabetes Quality Measures

	Nephropathy Screening[a]		Statin Therapy[b]		Statin Adherence[c]		HgbA1c Annual Measurement[d]		HgbA1c, poorly controlled[e]	
Variable	Odds Ratio (95% CI)	p value	Odds Ratio (95% CI)	p value	Odds Ratio (95% CI)	p value	Odds Ratio (95% CI)	p value	Odds Ratio (95% CI)	p value
CRH Intensity (ref = Low)										
Medium	0.83 (0.60 - 1.14)	0.25	1.04 (0.93 - 1.16)	0.46	0.93 (0.84 - 1.02)	0.14	0.94 (0.66 - 1.35)	0.75	0.92 (0.84 - 1.00)	0.05
High	0.79 (0.57 - 1.10)	0.16	1.04 (0.93 - 1.17)	0.47	0.92 (0.83 - 1.02)	0.12	0.96 (0.67 - 1.39)	0.84	0.92 (0.84 - 1.00)	0.06
Age	1.02 (1.01 - 1.03)	0.00	1.01 (1.00 - 1.01)	0.04	1.01 (1.01 - 1.02)	0.00	0.97 (0.95 - 0.98)	0.00	0.96 (0.96 - 0.96)	0.00
Sex (ref = Male)										
Female	0.57 (0.34 - 0.95)	0.03	0.52 (0.44 - 0.62)	0.00	0.79 (0.67 - 0.93)	0.01	0.88 (0.41 - 1.91)	0.75	0.74 (0.64 - 0.86)	0.00
Race and ethnicity[f] (ref Non-Hispanic White)										
Non-Hispanic Black	1.28 (0.88 - 1.86)	0.20	1.16 (1.02 - 1.31)	0.02	0.63 (0.57 - 0.70)	0.00	0.79 (0.53 - 1.17)	0.23	1.26 (1.15 - 1.38)	0.00
Hispanic	1.27 (0.71 - 2.25)	0.42	1.08 (0.89 - 1.31)	0.43	0.74 (0.63 - 0.87)	0.00	2.72 (1.00 - 7.40)	0.05	1.14 (0.99 - 1.31)	0.07
Asian/Pacific Islander/Native Hawaiian	1.40 (0.57 - 3.47)	0.46	1.43 (1.00 - 2.03)	0.05	0.71 (0.55 - 0.93)	0.01	0.46 (0.23 - 0.92)	0.03	1.17 (0.93 - 1.47)	0.18
American Indian/Alaska Native	1.21 (0.38 - 3.85)	0.75	0.75 (0.53 - 1.06)	0.10	0.59 (0.43 - 0.80)	0.00	2.42 (0.34 - 17.40)	0.38	1.24 (0.94 - 1.64)	0.13
Multiracial or other	1.61 (0.59 - 4.38)	0.35	0.80 (0.62 - 1.02)	0.08	0.80 (0.63 - 1.02)	0.07	1.45 (0.46 - 4.58)	0.53	0.91 (0.73 - 1.14)	0.42
Marital Status (ref = not married)										
Married	0.73 (0.56 - 0.96)	0.02	0.96 (0.88 - 1.05)	0.36	1.31 (1.21 - 1.42)	0.00	0.77 (0.57 - 1.05)	0.10	0.78 (0.73 - 0.84)	0.00
Rurality (ref = urban)										
Rural	0.85 (0.64 - 1.12)	0.24	0.95 (0.86 - 1.04)	0.28	1.13 (1.03 - 1.24)	0.01	1.08 (0.79 - 1.47)	0.65	0.89 (0.82 - 0.96)	0.00
Highly rural or insular islands	0.59 (0.33 - 1.04)	0.07	0.96 (0.78 - 1.19)	0.71	1.00 (0.82 - 1.21)	0.97	1.35 (0.62 - 2.95)	0.45	0.89 (0.75 - 1.06)	0.19
Drive distance to PCC	1.01 (1.00 - 1.02)	0.00	1.00 (1.00 - 1.00)	0.80	1.00 (1.00 - 1.00)	0.72	1.01 (1.00 - 1.02)	0.17	1.00 (1.00 - 1.00)	0.00
Number of primary care visits	1.04 (1.01 - 1.08)	0.01	1.03 (1.02 - 1.04)	0.00	1.00 (0.99 - 1.00)	0.20	1.06 (1.02 - 1.10)	0.00	1.04 (1.04 - 1.05)	0.00
Gagne Comorbidity Score[g]	1.03 (0.92 - 1.15)	0.62	0.99 (0.96 - 1.02)	0.37	0.98 (0.95 - 1.00)	0.06	0.92 (0.84 - 1.02)	0.10	1.00 (0.98 - 1.02)	0.97
Nosos Comorbidity Score[h]	5.02 (3.59 - 7.03)	0.00	1.24 (1.16 - 1.32)	0.00	1.14 (1.09 - 1.21)	0.00	2.86 (2.10 - 3.90)	0.00	0.90 (0.87 - 0.94)	0.00
Neighborhood SES Index[i]	0.94 (0.89 - 0.99)	0.02	1.01 (0.99 - 1.03)	0.38	1.02 (1.00 - 1.04)	0.01	0.93 (0.88 - 0.98)	0.01	0.97 (0.96 - 0.99)	0.00

Abbreviations: HgbA1c, hemoglobin A1c; PCC, primary care clinic; SES, socioeconomic status

[a] Veterans with documented screening for nephropathy within measurement year

[b] Veterans with at least one dispensing of statin of any intensity within measurement year

[c] Veterans who have a statin prescribed for 80% of treatment period within measurement year

[d] Veterans with documentation of annual measurement of HgbA1c

[e] Veterans with most recent HgbA1c greater than 9 or no evidence of test within measurement year[f] Based on race and ethnicity categories developed by Hernandez et al[1]

[g] Scores range from <0 to >9, with increased scores corresponding to increased risk of 1-year mortality[2]

© 2025 Liu T et al. *JAMA Network Open.*

[h] The Nosos scores are centered around 1, which indicates the patient is expected to have costs that are the national average for VA patients. If a patient has a risk score of 2.5, then the patient has an expected cost that is 2.5 times higher than the average VA patient.[3,4]
[i] Neighborhood socioeconomic status is reported as deciles based on census data, as a surrogate marker for income

© 2025 Liu T et al. *JAMA Network Open.*

eTable 6. Multivariable Regression Results of Association Between Clinical Resource Hub (CRH) Intensity and Hypertension Quality Measures

	BP, well controlled (DM)[a]		BP, well controlled (HTN)[b]	
Variable	Odds Ratio (95% CI)	p value	Odds Ratio (95% CI)	p value
CRH Intensity (ref = Low)				
Medium	1.08 (1.00 - 1.17)	0.05	1.04 (0.98 - 1.10)	0.16
High	1.18 (1.09 - 1.28)	0.00	1.16 (1.10 - 1.23)	0.00
Age	0.99 (0.99 - 0.99)	0.00	1.00 (1.00 - 1.00)	0.23
Sex (ref = Male)				
Female	0.98 (0.86 - 1.13)	0.83	0.98 (0.89 - 1.08)	0.66
Race and ethnicity[c] (ref Non-Hispanic White)				
Non-Hispanic Black	0.72 (0.66 - 0.79)	0.00	0.83 (0.78 - 0.88)	0.00
Hispanic	0.92 (0.80 - 1.05)	0.21	1.02 (0.92 - 1.14)	0.66
Asian/Pacific Islander/Native Hawaiian	0.97 (0.77 - 1.22)	0.80	1.24 (1.03 - 1.50)	0.02
American Indian/Alaska Native	0.98 (0.74 - 1.29)	0.87	0.93 (0.75 - 1.14)	0.46
Multiracial or other	0.97 (0.79 - 1.18)	0.74	1.11 (0.95 - 1.29)	0.19
Marital Status (ref = not married)				
Married	1.16 (1.09 - 1.23)	0.00	1.11 (1.06 - 1.16)	0.00
Rurality (ref = urban)				
Rural	0.99 (0.92 - 1.07)	0.86	1.02 (0.97 - 1.07)	0.42
Highly rural or insular islands	1.08 (0.93 - 1.27)	0.31	1.01 (0.90 - 1.12)	0.91
Drive distance to PCC	1.00 (1.00 - 1.00)	0.03	1.00 (1.00 - 1.00)	0.62
Number of primary care visits	1.01 (1.00 - 1.01)	0.01	1.01 (1.00 - 1.01)	0.00
Gagne Comorbidity Score[d]	1.02 (1.00 - 1.04)	0.04	1.01 (0.99 - 1.02)	0.29
Nosos Comorbidity Score[e]	0.96 (0.93 - 1.00)	0.03	1.04 (1.01 - 1.07)	0.02
Neighborhood SES Index[f]	1.01 (0.99 - 1.02)	0.29	1.00 (0.99 - 1.01)	0.91

Abbreviations: BP, Blood pressure; DM, diabetes mellitus; HTN, hypertension; PCC, primary care clinic; SES, socioeconomic status

[a] Veterans with diabetes mellitus with most recent recorded BP as < 140/90mmHg
[b] Veterans with hypertension with most recent recorded BP as < 140/90mmHg
[c] Based on race and ethnicity categories developed by Hernandez et al[1]
[d] Scores range from <0 to >9, with increased scores corresponding to increased risk of 1-year mortality[2]
[e] The Nosos scores are centered around 1, which indicates the patient is expected to have costs that are the national average for VA patients. If a patient has a risk score of 2.5, then the patient has an expected cost that is 2.5 times higher than the average VA patient.[3,4]
[f] Neighborhood socioeconomic status is reported as deciles based on census data, as a surrogate marker for income[5]

© 2025 Liu T et al. *JAMA Network Open.*

eReferences

1. Hernandez SE, Sylling PW, Mor MK, et al. Developing an Algorithm for Combining Race and Ethnicity Data Sources in the Veterans Health Administration. *Mil Med*. 2020;185(3-4):e495-e500. doi:10.1093/milmed/usz322
2. Gagne JJ, Glynn RJ, Avorn J, Levin R, Schneeweiss S. A combined comorbidity score predicted mortality in elderly patients better than existing scores. *J Clin Epidemiol*. 2011;64(7):749-759. doi:10.1016/j.jclinepi.2010.10.004
3. Yoon J, Chow A. Comparing chronic condition rates using ICD-9 and ICD-10 in VA patients FY2014–2016. *BMC Health Serv Res*. 2017;17(1):572. doi:10.1186/s12913-017-2504-9
4. Wagner T, Moran E, Shen M, Gehlert E. HERC's Guide to the Nosos Risk Adjustment Score. May 2024. Accessed June 14, 2024. https://www.herc.research.va.gov/include/page.asp?id=guidebook-nosos.
5. Nelson K, Schwartz G, Hernandez S, Simonetti J, Curtis I, Fihn SD. The Association Between Neighborhood Environment and Mortality: Results from a National Study of Veterans. *J Gen Intern Med*. 2017;32(4):416-422. doi:10.1007/s11606-016-3905-x

© 2025 Liu T et al. *JAMA Network Open.*
