# Supplementary material for: National Telehealth Contingency Staffing Program and Primary Care Quality in the VHA
Source: JAMA Netw Open. 2025 Jan 7;8(1):e2453324. doi: 10.1001/jamanetworkopen.2024.53324 (PMC11707631; doi:10.1001/jamanetworkopen.2024.53324)
Supplement: Supplement 2. — Data Sharing Statement [file jamanetwopen-e2453324-s002.pdf]

Data Sharing Statement

Liu. National Telehealth Contingency Staffing Program and Primary Care Quality in the VHA. *JAMA Netw Open*. Published January 07, 2025. doi:10.1001/jamanetworkopen.2024.53324

Data
Data available: No

Additional Information
Explanation for why data not available: Due to US Department of Veterans Affairs (VA) regulations and our ethics agreements, the analytic data sets used for this study are not permitted to leave the VA firewall without a Data Use Agreement. This limitation is consistent with other studies based on VA data. However, VA data are made freely available to researchers with an approved VA study protocol. For more information, please visit https://www.virec.research.va.gov or contact the VA Information Resource Center at VIReC@va.gov.
